# Biosynthesis and metabolic engineering of 1-hydroxyphenazine in *Pseudomonas chlororaphis* H18

**DOI:** 10.1186/s12934-021-01731-y

**Published:** 2021-12-30

**Authors:** Yupeng Wan, Hongchen Liu, Mo Xian, Wei Huang

**Affiliations:** 1 CAS Key Lab of Biobased Materials, Qingdao Institute of Bioenergy and Bioprocess Technology, Chinese Academy of Sciences, Qingdao, 266101 China; 2 University of Chinese Academy of Sciences, Beijing, 100049 China

**Keywords:** 1-Hydroxyphenazine, Biosynthesis, Metabolic engineering, *Pseudomonas chlororaphis* H18

## Abstract

**Background:**

1-Hydroxyphenazine (1-OH-PHZ) is a phenazine microbial metabolite with broad-spectrum antibacterial activities against a lot of plant pathogens. However, its use is hampered by the low yield all along. Metabolic engineering of microorganisms is an increasingly powerful method for the production of valuable organisms at high levels. *Pseudomonas chlororaphis* is recognized as a safe and effective plant rhizosphere growth-promoting bacterium, and faster growth rate using glycerol or glucose as a renewable carbon source. Therefore, *Pseudomonas chlororaphis* is particularly suitable as the chassis cell for the modification and engineering of phenazines.

**Results:**

In this study, enzyme PhzS (monooxygenase) was heterologously expressed in a phenazine-1-carboxylic acid (PCA) generating strain *Pseudomonas chlororaphis* H18, and 1-hydroxyphenazine was isolated, characterized in the genetically modified strain. Next, the yield of 1-hydroxyphenazine was systematically engineered by the strategies including (1) semi-rational design remodeling of crucial protein PhzS, (2) blocking intermediate PCA consumption branch pathway, (3) enhancing the precursor pool, (4) engineering regulatory genes, etc. Finally, the titer of 1-hydroxyphenazine reached 3.6 g/L in 5 L fermenter in 54 h.

**Conclusions:**

The 1-OH-PHZ production of *Pseudomonas chlororaphis* H18 was greatly improved through systematically engineering strategies, which is the highest, reported to date. This work provides a promising platform for 1-hydroxyphenazine engineering and production.

**Graphical Abstract:**

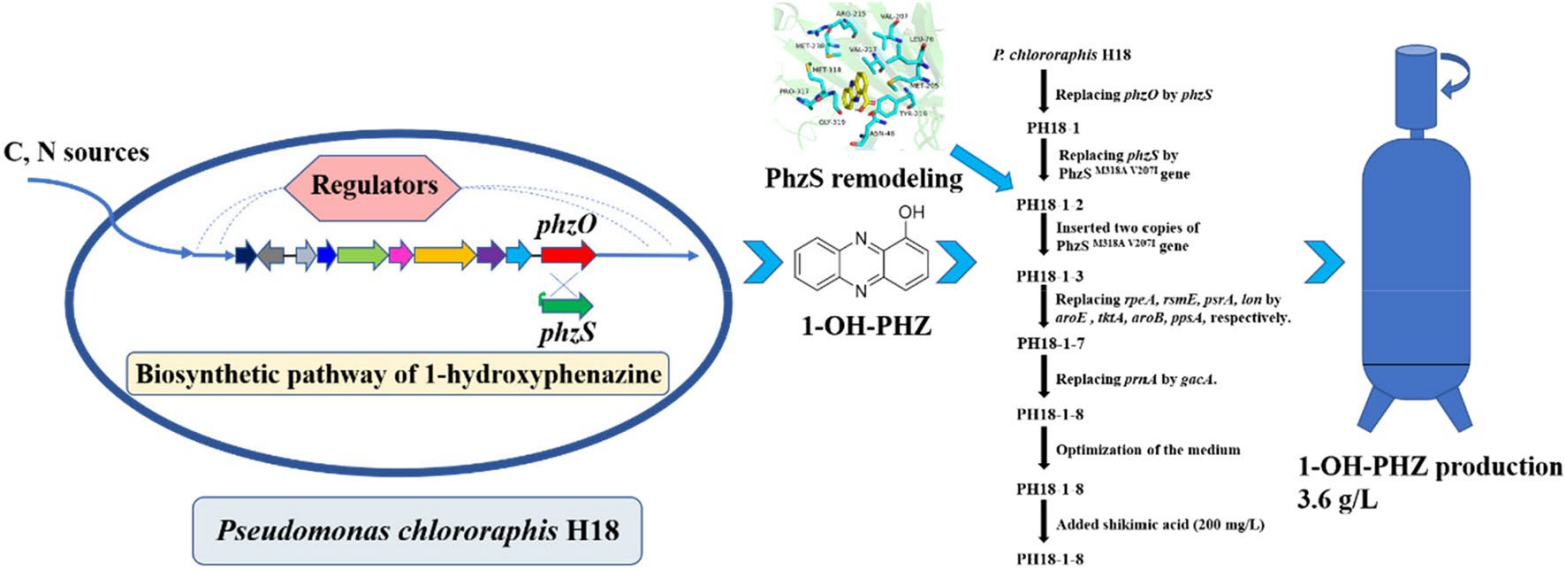

**Supplementary Information:**

The online version contains supplementary material available at 10.1186/s12934-021-01731-y.

## Background

Phenazines are a large class of nitrogen-containing heterocyclic naturally or chemically synthesized colored aromatic products [1, 2]. As the secondary metabolites produced by bacteria and archaea, they are used for the biological control of plant pathogens based on their broad-spectrum antifungal activity, minimal toxicity to humans, animals, and the environment [3, 4]. One of the important natural phenazine compounds, phenazine-1-carboxylic acid (PCA, **1**), has been developed as a new biopesticide “Shenqinmycin” in 2011 and is widely used [5, 6]. Although chemical synthesis of phenazines is feasible [7], the conversion rate is low, and the organic solvent or toxic byproducts in the reaction process may cause environmental pollution problems. Biosynthetic manufacturing of high-value chemicals such as antibiotics, biofuels, or biopolymers from easily obtained renewable feedstock is an efficient, safe, and green production method. So far, there have been more than 150 reported phenazine natural products [8]. Most naturally occurring phenazines’ yield is extremely low, and only a handful of phenazine derivatives, such as PCA [5], 2-hydroxyphenazine (2-OH-PHZ, **2**) [9], and phenazine-1-carboxamide (PCN) [10] have been explored to enhance the fermentation performance. Therefore, research on the improving of phenazine production is urgently needed.

**Fig. 1:**
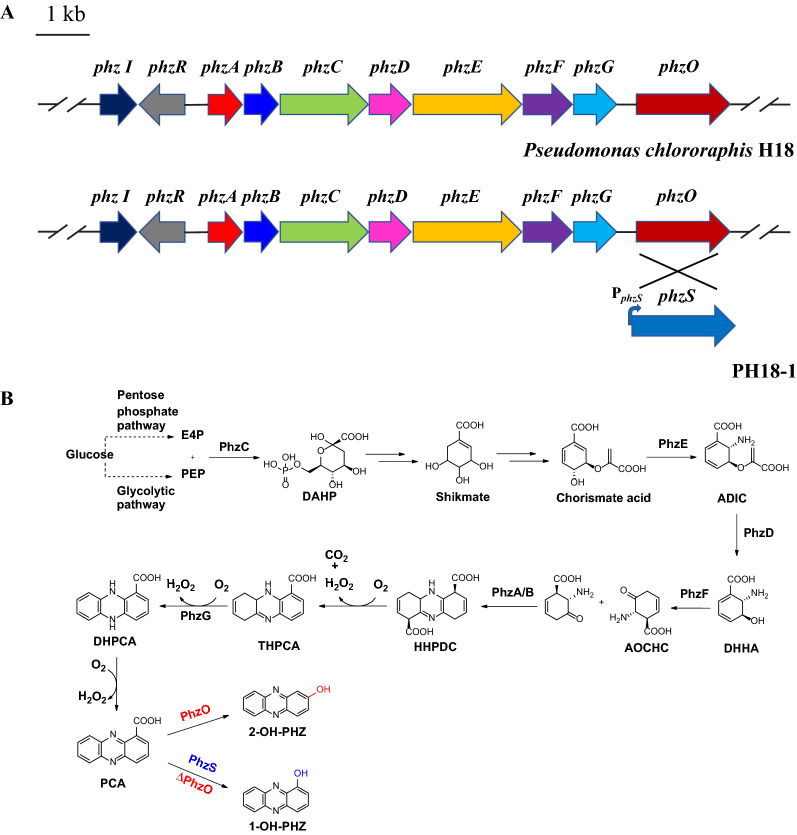
**A** The biosynthetic gene cluster (9.8 kb) of phenazine compounds in *P. chlororaphis* H18. **B** The biosynthetic pathway of 1-hydroxyphenazine

1-Hydroxyphenazine (1-OH-PHZ) is a phenazine family compound separated from *Pseudomonas aeruginosa*, *Pseudomonas monteilii*, *Nocardiopsis dassonvillei* (1.0 mg extracted from 96 L fermentation medium), and *Streptomyces* sp. (30 mg extracted from 20 L fermentation medium) [11–13]. Previous studies showed that it possesses antibacterial, antifungal, and anti-cancer activity. Due to the low fermentation yield and the lack of diversity, its industrial application potential is restricted. Recently, we screened a strain *Pseudomonas chlororaphis* H18 in the natural plant rhizosphere, which was confirmed to produce phenazine-1-carboxylic acid (PCA) and 2-hydroxyphenazine (2-OH-PHZ). The titers of both compounds are higher than most of the known wild type *Pseudomonas* strains. Through genome sequencing and protein sequence alignment, the biosynthetic gene cluster of PCA in *P. chlororaphis* H18 was located. The core loci *phzABCDEFGO* is responsible for PCA and 2-OH-PHZ biosynthesis (Fig. 1A) [14–21], and PhzABCDEFGO has high similarity to those proteins in the other PCA, 2-OH-PHZ producing strains *Pseudomonas chlororaphis* GP72 [22] (Additional file 1: Table S3), *Pseudomonas chlororaphis* LX24 [23]*, or Pseudomonas chlororaphis* Qlu-1 [24]. As the basic biosynthetic route of phenazines has been elucidated, researchers can use combinatorial biosynthesis to obtain new derivatives [25]. *Pseudomonas chlororaphis* is recognized as a safe and effective plant rhizosphere growth-promoting bacterium [26], therefore we decided to adopt *P. chlororaphis* H18 as the chassis cell for the modification and engineering of phenazines.

In this study, a heterologous monooxygenase PhzS from *P. aeruginosa* PAO1 [27] was expressed in *P. chlororaphis* H18 which led to the production of 1-OH-PHZ. Afterwards, the yield of 1-OH-PHZ was optimized via a variety of strategies, which reaches the highest as far as we know.

## Results and discussion

### Design of 1-OH-PHZ biosynthetic pathway in *P. chlororaphis* H18

We have isolated a strain *P. chlororaphis* H18 that can secrete reddish-brown pigments from the plant rhizosphere soil, and its colorful metabolites were purified and their structures elucidated to be PCA (**1**), 2-OH-PHZ (**2**), and 2-hydroxy-phenazine-1-carboxylic acid (**3**). Using genome-mining and sequence alignment, their biosynthetic gene cluster was confirmed (Fig. 1A). Since the basic biosynthetic pathway of phenazines has already been clearly resolved, proteins PhzABCDEFGO catalyze the shikimate pathway end product chorismate to form the phenazine common precursor PCA (Fig. 1B). Then, a monooxygenase PhzO converts PCA into 2-OH-PHZ, and 2-hydroxy-phenazine-1-carboxylic acid is the intermediate of this process [20]. A previous study showed that FAD-dependent monooxygenase PhzS of *P. aeruginosa* PAO1 can catalyze the oxidative decarboxylation of PCA to generate 1-OH-PHZ [28]. So we planned to integrate this *phzS* gene into *P. chlororaphis* H18 genome to construct 1-OH-PHZ biosynthetic pathway.

### Biosynthesis and identification of 1-OH-PHZ

**Fig. 2:**
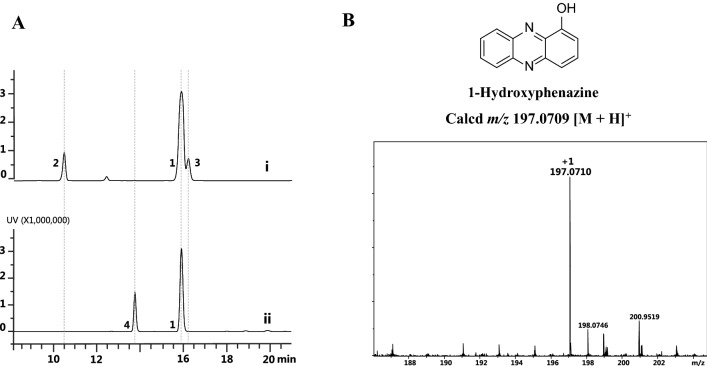
1-Hydroxyphenazine producing strain fermentation and detection. **A** HPLC chromatograms of fermentation broth of (i) *P. chlororaphis* H18, (ii) *P. chlororaphis* H18-1. **B** The HRMS analysis of 1-hydroxyphenazine. **1**, phenazine-1-carboxylic acid; **2**, 2-hydroxyphenazine; **3**, 2-hydroxy-phenazine-1-carboxylic acid; **4**, 1-hydroxyphenazine

When cultured in KB medium for 48 h, *P. chlororaphis* H18 produces 86.6 mg/L PCA, 71.8 mg/L 2-OH-PHZ, and 14.8 mg/L 2-hydroxy-phenazine-1-carboxylic acid. The formation of 2-OH-PHZ and 2-hydroxy-phenazine-1-carboxylic acid catalyzed by PhzO consumed a significant amount of PhzS substrate PCA. Therefore, we synthesized gene *phzS* from *P. aeruginosa* PAO1 and replaced *phzO* with homologous recombination to obtain mutant PH18-1 (Additional file 1: Figs. S3 and S4). In the PH18-1 fermentation broth, 72.3 mg/L of 1-OH-PHZ (**4**) generation was detected and the production of 2-OH-PHZ (**2**) and 2-hydroxy-phenazine-1-carboxylic acid (**3**) was abolished (Fig. 2A, B). 1-OH-PHZ shows outstanding biological activities against crop pathogens, for instance, *Bipolaris maydis*, *Alternaria* *solani*, and *Fusarium* *graminearum* (Additional file 1: Table S5).

### Semi-rational design and remodeling to improve the catalytic activity of PhzS

**Fig. 3:**
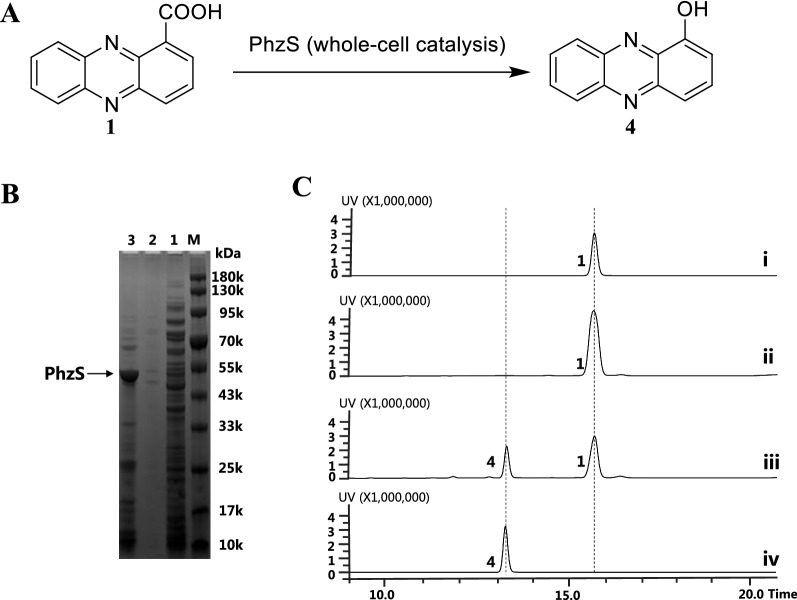
The protein expression of PhzS and enzymatic catalytic reactions in vitro. **A** Schematic representation of PCA oxidative decarboxylation by PhzS. **B** SDS-PAGE of PhzS (43 kD). M, marker; 1, unbound proteins; 2, washed proteins with 20 mM of imidazole; and 3, eluted proteins with 300 mM of imidazole. **C** HPLC chromatograms of PhzS catalyzed reaction with PCA as substrate at 250 nm. Reaction conditions: phenazine-1-carboxylic acid (0.5 mM), NADH (50 μM), PhzS whole-cell in 50 mM HEPES (pH 7.5) at 30 °C for 1 h. (i) phenazine-1-carboxylic acid standard; (ii) Boiled PhzS whole-cell and NADH with phenazine-1-carboxylic acid; (iii) PhzS whole-cell and NADH with phenazine-1-carboxylic acid; (iv) 1-hydroxyphenazine standard. **1**, phenazine-1-carboxylic acid; **4**, 1-hydroxyphenazine

**Fig. 4:**
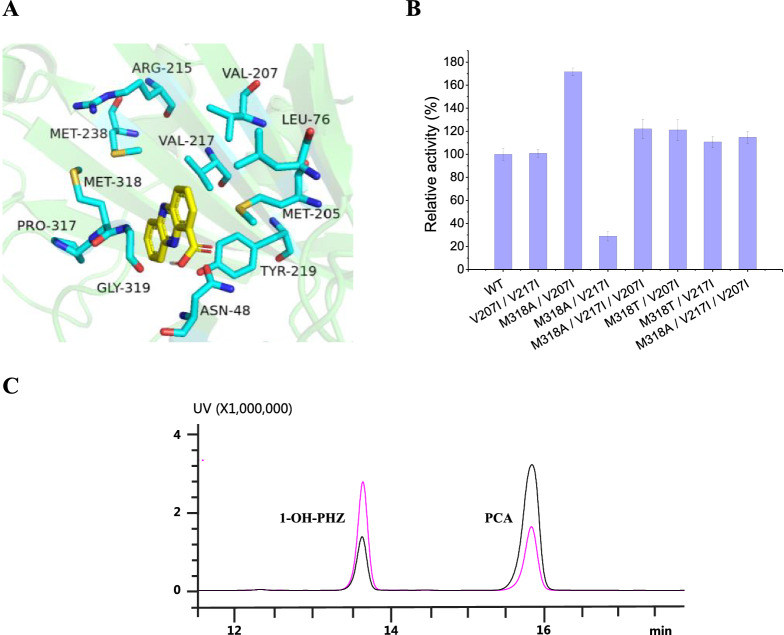
Rational design of the PhzS to improve its activity to PCA. **A** Docking of the ligand PCA into the PhzS crystal structure. PCA is shown in yellow. **B** Relative activity of wild type PhzS and mutants to PCA. **C** HPLC chromatograms of fermentation broth of PH18-1 and PH18-1–2 at 250 nm. The pink line: PH18-1–2; the black line: PH18-1. The data shown in B are from three experiment replicates, and are expressed as the mean value ± SD

The concentration of 1-OH-PHZ in PH18-1 fermentation broth is relatively low (72.3 mg/L), which limited its industrial application. To address this issue, the promotion of the 1-OH-PHZ conversion rate is an essential job. We know that enzymes are the most critical factors in the biosynthesis process of natural products, especially those rate-limiting step enzymes. In the 1-OH-PHZ biosynthetic pathway, the flavin-dependent hydroxylase PhzS catalyzes the crucial step of decarboxylation and hydroxylation at PCA 1-position. We believe that optimizing PhzS will be an effective way to improve pathway efficiency. Semi-rational design and molecular remodeling are feasible methods for promoting protein catalytic performance. First, PCA was docked into the crystal structure of PhzS (PDB: 2RGJ) as a ligand to predict the PCA-binding hydrophobic pocket or activity site dominated by N48, L76, M205, V207, R215, V217, Y219, M238, P317, M318, G319 [21] (Fig. 4A). To facilitate the detection of PhzS mutants’ activity, we expressed PhzS protein in *E. coli* BL21 Rosetta (DE3) and verified its activity by whole-cell catalysis (Fig. 3). Then, alanine scanning was employed to identify the function of these residues by detecting mutant enzyme activities on PCA in vitro. N48A, L76A, Y219A, and M238A mutants completely lost their catalytic abilities, only M318A presented a 24% activity increase. Next, amino acids M318 and V217 were subjected to saturation mutagenesis, M318T, M318I, and V217I resulted in 40%, 5%, and 35% higher activity than the wild type enzyme (Additional file 1: Fig. S1). More site-directed mutants in M205, V207, R215, P317, and G319 were constructed using residues I, S, T, and V respectively, among which better activity (1.4-fold of the WT strain) was observed in PhzS^V207I^ (Additional file 1: Fig. S2). Afterward, we used M318A, M318T, V217I, and V207I to construct double and triple mutants. In these mutants, the resulting PhzS^M318A/V207I^ achieved the highest reactivity (an increase of 70% compared with PhzS) to catalyze PCA (Fig. 4B). These mutations are likely to remodel PhzS conformation and consequently influence the substrate-binding pocket, which enhances PhzS catalytic activity. When protein PhzS^M318A/V207I^ was introduced into *P. chlororaphis* H18, the obtained strain PH18-1–2 presented 151.8 mg/L 1-OH-PHZ concentration, which is 1.1 times higher than PH18-1 (Fig. 4C). This indicates that the engineering of PhzS is an effective way to increase the fermentation yield of 1-OH-PHZ.

The PH18-1–2 fermentation result showed that there is still a certain amount of precursor PCA in the fermentation broth. Afterward, we try to enhance the conversion of PCA to 1-OH-PHZ by adding PhzS copy numbers. When we inserted two copies of the PhzS ^M318A/V207I^ gene to replace PhzS in PH18-1, the constructed strain PH18-1–3 produced 202.4 mg/L 1-OH-PHZ and more PCA is consumed.

### Disruption of negative regulatory genes and enrichment of precursor supply to enhance 1-OH-PHZ production

**Fig. 5:**
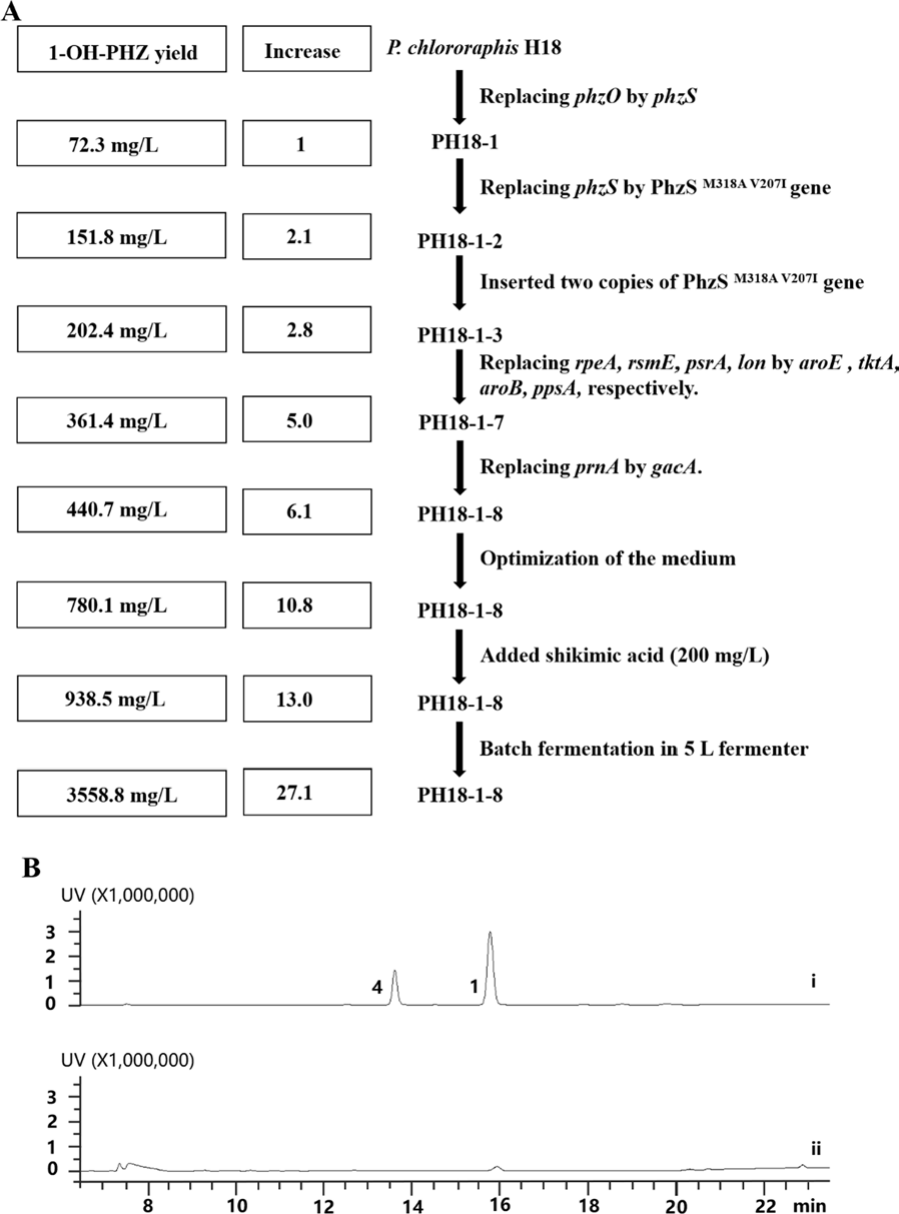
The strategies for increasing 1-hydroxyphenazine production. **A** A summary of the steps in the genetic and metabolic engineering of *P. chlororaphis* H18 for 1-OH-PHZ production. **B** HPLC chromatograms of fermentation broth of PH18-1 and knocking out *gacA* gene in *P. chlororaphis* H18 at 250 nm. (i) PH18-1 fermentation broth; (ii) Fermentation broth of knocking out *gacA* gene in *P. chlororaphis* H18. **1**, phenazine-1-carboxylic acid; **4**, 1-hydroxyphenazine

In order to improve 1-OH-PHZ yield, we first chose to increase the supply of phenazine substrate pool in *P. chlororaphis* H18. Chorismate, a shikimate pathway end product is the key phenazine derivative precursor. Several enzymes such as phosphoenolpyruvate (PEP) synthetase PpsA, transketolase TktA, quinate/shikimate dehydrogenase AroE, and dehydroquinic acid synthase AroB have been studied to enhance the shikimate pathway and chorismate [9, 24]. Through sequence alignment, we found that, PpsA, TktA, AroB, and AroE homologous proteins genes all exist in the in *P. chlororaphis* H18 genome. Hence, we planned to introduce one more copy of *ppsA*, *tktA*, *aroB*, and *aroE* genes into *P. chlororaphis* H18. Besides, RpeA, RsmE, PsrA, and Lon have been reported as negative regulators in *Pseudomonas chlororaphis* strains [29–32]. The disruption of these regulatory genes has been proven to effectively improve phenazines titer. Thus, we cloned *aroE*, *tktA*, *aroB*, and *ppsA* genes from *P. chlororaphis* H18 to replace the possible negative regulatory genes *repA*, *rsmE*, *psrA*, and *lon* to obtain strains PH18-1-4, PH18-1-5, PH18-1-6, PH18-1-7 respectively (Fig. 5A). According to the HPLC detection results, the 1-OH-PHZ concentration in the constructed final strain PH18-1-7 was 361.4 mg/L, a fourfold increase over PH18-1 when fermented in KB medium.

### Further improvement of 1-OH-PHZ production

**Fig. 6:**
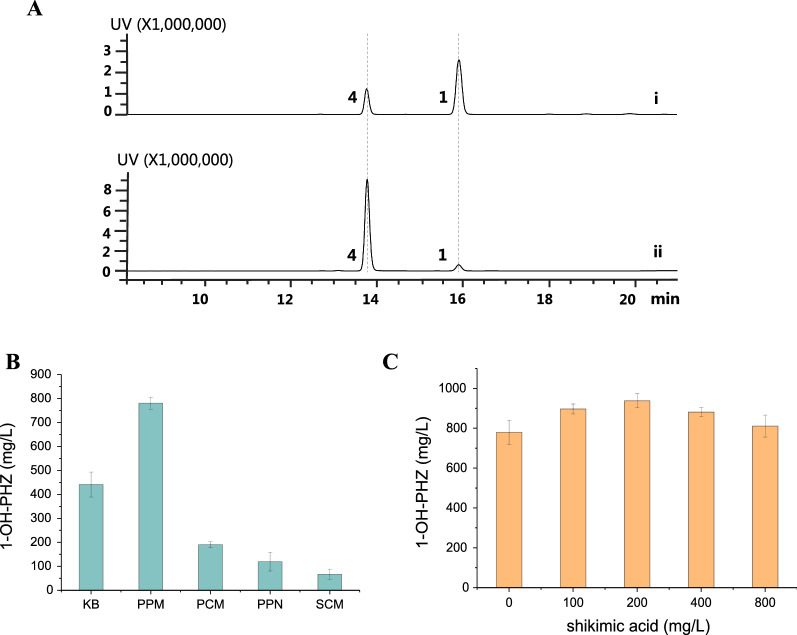
Improvement of 1-OH-PHZ production. **A** HPLC chromatograms of fermentation broth of PH18-1 and PH18-1–8 at 250 nm. (i) Fermentation broth of PH18-1; (ii) Fermentation broth of PH18-1–8. **1**, phenazine-1-carboxylic acid; **4**, 1-hydroxyphenazine. **B** PH18-1–8 production of 1-hydroxyphenazine in different media. **C** The yield of PH18-1–8 1-hydroxyphenazine by adding different concentrations of shikimic acid to the medium. The data are from three experiment replicates, and are expressed as the mean value ± SD

Research suggested that GacA is a positive global regulator for phenazine biosynthesis in *Pseudomonas chlororaphis* 30–84 [32], but negatively regulated phenazines production in *Pseudomonas* sp. M18 and *Pseudomonas chlororaphis* GP72 [33]*.* When we knocked out the *gacA* gene from *P. chlororaphis* H18, the phenazine generation was almost abolished, which means GacA plays a role in positive regulation in *P. chlororaphis* H18 on PCA synthesis (Fig. 5B). Afterward, an additional copy of *gacA* was introduced into PH18-1–7 to replace the secondary metabolite pyrrolnitrin biosynthetic gene *prnA.* The resultant mutant PH18-1–8 had a 20% increase in 1-OH-PHZ titer (Fig. 5A and Additional file 1: Fig. S4), which is fivefold more than mutant PH18-1 (Fig. 6A).

To further optimize 1-OH-PHZ titer, we tried different kinds of fermentation medium, for instance, KB, PPM, PCM, PPN, and SCM. The HPLC detection data illustrated that PPM is the best for 1-OH-PHZ production, in which the 1-OH-PHZ level is 70% higher than in KB broth (Fig. 6B).

Moreover, shikimic acid is a chief precursor of chorismate in the shikimate pathway, and all known phenazines are converted from chorismate. Hence, we suppose that directly increasing the supply of upstream intermediate or precursor substrate may affect the enhancement of 1-OH-PHZ production. Given the high price of chorismate on the market, the economic shikimic acid is suitable for feeding. When 100 mg/L, 200 mg/L, 400 mg/L, or 800 mg/L of shikimic acid was fed into 50 mL PH18-1–8 strain fermentation broth respectively in 12 h. HPLC detection demonstrates that the addition of shikimic acid can indeed improve the 1-OH-PHZ level (Fig. 6C). Among them, 200 mg/L shikimic acid feeding showed the best effect (20% increase over no feeding). Although higher concentrations of shikimic acid can also increase 1-OH-PHZ titer, the burden of metabolism or feedback inhibition of the synthetic pathway probably influences the yield. Otherwise, engineering feedback resistance of DHAP synthase is another effective approach to improve the yield of shikimate derived products, which could be used in our future work.

### Optimization of fed-batch 1-OH-PHZ fermentation

**Fig. 7:**
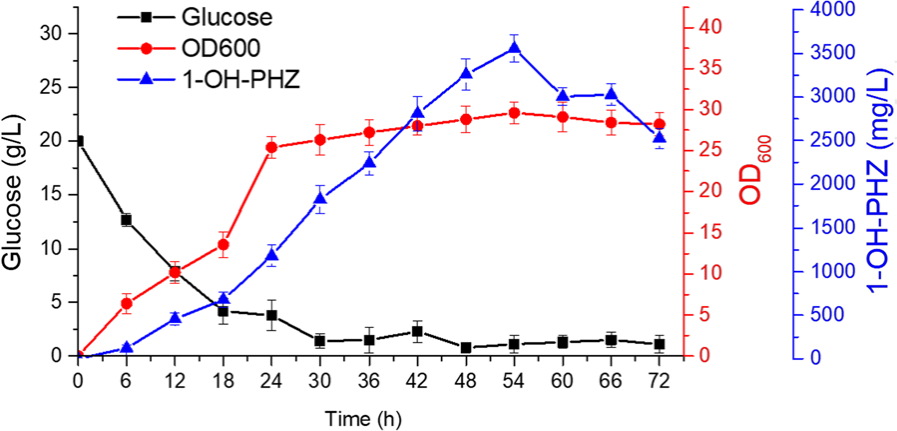
Production of 1-hydroxyphenazine by *P. chlororaphis* H18-1–8 via fed-batch fermentation. Time course of cell growth, glucose consumption and 1-hydroxyphenazine production during fed-batch fermentation. The data are from three experiment replicates, and are expressed as the mean value ± SD

To combine all the above engineering modifications, mutant strain PH18-1-8 (Fig. 5A and Additional file 1: Fig. S4) could generate 938.5 mg/L of 1-OH-PHZ in 50 mL shake flasks. Further improvement of the 1-OH-PHZ production level was carried out by fed-batch fermentation. Strain PH18-1-8 was first employed in a 5 L fermenter containing 2 L PPM liquid medium. During the fermentation process, glucose was also added to meet the demand of the carbon source. Production of 1-OH-PHZ continued to increase until 54 h, and the highest production was 3.6 g/L. The time-course analysis of glucose consumption, OD value, and 1-OH-PHZ concentration during fermentation are illustrated in Fig. 7, respectively.

## Conclusions

In this study, we screened a PCA and 2-OH-PHZ producing strain *P. chlororaphis* H18. Using it as the chassis cell, 1-OH-PHZ was generated by introducing a monooxygenase PhzS. Then, we improved the 1-OH-PHZ fermentation level through different methods, including (1) semi-rational design remodeling of critical enzyme PhzS, (2) blocking 2-hydroxyphenazine synthesis to reduce the consumption of intermediate PCA, (3) enhancing the precursor pool by adding copy numbers of shikimate pathway genes or directly feeding shikimic acid into the fermentation system, (4) engineering regulatory genes, etc. The 1-OH-PHZ concentration of the final engineered strain PH18-1-8 reached 938.5 mg/L in 50 mL shake flasks, which is 12 times more than the original strain PH18-1. Finally, through fed-batch optimization, the titer of 1-hydroxyphenazine reached 3.6 g/L in a 5 L fermenter in 54 h, which is the highest reported to date. This research paved a way for the further combinatorial biosynthesis and metabolic engineering of 1-hydroxyphenazine and its derivatives.

## Materials and methods

### Bacterial strains, plasmids, primers, culture media, and cultivation conditions.

The strains and Plasmids used in this work are indicated in Additional file 1: Table S1. *Escherichia coli* DH5α, BL21 (DE3), Rosetta (DE3) and S17-1 were cultured at 37 °C in a Luria–Bertani (LB) medium. *P. chlororaphis* H18 and mutants were grown in King’s B (KB) media. For fermentation, one colony of *P. chlororaphis* H18 was picked from the KB plate and inoculated into 3 mL of the KB liquid medium as a seed culture at 30 °C with 200 rpm of shaking overnight. Then, the seed culture (1 mL inoculum) was inoculated into 50 mL KB liquid medium in 250 mL flasks at 30 °C with 200 rpm for 24–72 h. When required, chloromycetin (Cm, 50 μg/mL) or kanamycin (Kan, 100 μg/mL) was used in the media. The production of 1-hydroxyphenazine was tested with different media (KB, PPM, PCM, PPN, SCM), PPM medium contained 22 g/L tryptone, 20 g/L glucose, 5 g/L KNO_3_, pH 7.5; PCM medium contained 30.0 g/L soybean meal, 7.5 g/L corn steep liquor, 10.0 g/L glucose, 10.0 g/L tryptone, 1.0 g/L MgSO_4_, 2.5 g/L KNO_3_, pH 7.5; PPN medium contained 22 g/L Tryptone, 20 g/L glucose, and 15 g/L KNO_3_, pH 7.5; SCM medium contained 65.02 g/L soybean meal, 15.36 g/L corn steep liquor, 12 g/L glucose, 21.70 mL/L ethanol, and 1 g/L MgSO_4_, pH 7.5. The DNA sequencing and Polymerase chain reaction (PCR) primers were performed by Tsingke Biotechnology Co.,Ltd. The genes were synthesized by Beijing Liuhe BGI Co.,Ltd. DNA extraction and kits for plasmid preparation were purchased from Omega.

### 16S rRNA sequencing and whole genome sequencing.

The 16 S rRNA sequence of *P. chlororaphis* H18 was obtained by amplification of primers 16 S rRNA (27F) and 16 S rRNA (1492R), the PCR product of which was sequenced by Tsingke BiotechnologyCo.,Ltd. The whole genome sequencing of *P. chlororaphis* H18 was performed by GENEWIZ Biotechnology Co., Ltd.

### Gene replacement procedure

Homologous recombination was used for gene replacement. The construction of plasmid included three fragments and pK18mob*sacB* vector. Primers F/R were used to amplify the fragment of overexpressed gene and its promotor using synthetic gene or total DNA of *P. chlororaphis* H18 as a template. Meanwhile, primers LF/LR and RF/RR were used to amplify the fragment of target gene upstream and downstream, respectively. Since the success rate of four-fragment ligation is too low, the plasmids were constructed step by step. Utilizing pK18mob*sacB* as a vector, it is connected to the downstream fragment of target gene and overexpressed gene through T4 ligase (NEB, USA), and then connected to the upstream fragment of the target gene. The correctly sequenced plasmid was introduced into S17-1 (λ pir) and transferred into *P. chlororaphis* H18 through conjugative transfer, and the target gene was replaced with an overexpressed gene by double crossover. Eventually, the successful replacement was verified by PCR.

### Point mutations in the *phzS* gene

PhzS mutants were constructed by the splicing-by-overlap-extension method. The sequences upstream and downstream of the mutation site were amplified first individually from pET30a-PhzS. The upstream of primer pairs were F1/R1 and the downstream of primer pairs were F2/R2. The PCR products of the above primer pairs were used as templates for another round of PCR using primers F1 and R2. The PCR products of the second round were digested with restriction enzymes BamHI/XhoI and ligated into the BamHI/XhoI sites of pET-30a containing an N-terminal 6xHis-tag. The mutations were verified by DNA sequencing.

### Separation of phenazine compounds

Fermentation was performed in 500 mL flasks containing 100 mL of the PPM medium and incubated for 3 days at 30 °C with 200 rpm for a total of 1 L. The broth was extracted with ethyl acetate three times and the extract was evaporated to dryness under vacuum at 40 °C. Chromatography column (5 × 20 cm) was packed with silica gel (50 g). The crude extract was dissolved in 50 mL of CH_3_OH and evaporated to dryness under vacuum at 40 °C with 3 g silica gel (aladdin, AR, 300–400 mesh) and applied uniformly onto the top of the column. Gradient elution of the column was carried out with mixtures of Petroleum ether, dichloromethane, and ethyl acetate in increasing order of polarity. Fractions of 150 mL each were collected and a total of 36 fractions were collected and pooled based on their TLC profile into 8 fractions. It was further purified by preparative thin-layer chromatography (PTLC) on pre-coated Silica Gel plates (HSGF254, 20 × 20 cm). The PTLC plates were developed in CH_2_Cl_2_. The desired band was scratched out of the PTLC plate after air-drying visualized under UV light (254 nm) and the metabolite was extracted with MeOH.

### Measurement and detection of phenazine compounds production

In order to extract the phenazine compounds, 5 mL of fermentation culture was mixed with 250 μL of diluted HCl and extracted three times using an equal volume of ethyl acetate with vigorous shaking. After centrifugation at 13,000×*g* for 10 min, the organic phase was taken into the bottle and detected with HPLC at 250 nm. The mobile phase detected by HPLC was acetonitrile and water containing 0.1% TFA. 3 μL of extracted phenazine compounds sample was taken for HPLC analysis (Nexera XR) under the following conditions: C18 reversed-phase column (Agilent 5 TC-C18 (2) (250 × 4.6 mm)) eluted with acetonitrile and water containing 0.1% TFA. 1-OH-PHZ production was quantified using peak area (A) in HPLC elute according to the following formula: 1-OH-PHZ (mg/L) = A/21,033.7–9.45, which was derived from a dose-peak area plot using purified 1-OH-PHZ with a correlation co-efficiency (R2) of 0.999. Liquid chromatography high-resolution mass spectrometry (LC-HRMS) analysis was performed on an Agilent Technologies 6520 Accurate-Mass Q-TOF LC–MS instrument with an Agilent Eclipse Plus C18 column (4.6 × 100 mm). LC detection procedure: 15% CH_3_CN in H_2_O from 0 to 5 min, 15% to 95% CH_3_CN in H_2_O from 5 to 12 min, 95% CH_3_CN in H_2_O from 12 to 15 min, 95% to 15% CH_3_CN in H_2_O from 15 to 18 min, 15% CH_3_CN in H_2_O from 18 to 20 min (H_2_O and CH_3_CN containing 0.1% formic acid (vol/vol)). The flow rate of 0.2 mL/min was used. Nuclear magnetic resonance (NMR) spectra were recorded on a Bruker Biospin 600 MHz spectrometer.

### In vitro enzymatic assays

The enzymatic activity of PhzS mutants was measured and the reaction mixture (100 μL) contained substrate (0.5 mM), NADH (50 μM), PhzS mutants whole-cell in 50 mM HEPES (pH 7.5) at 30 °C for 1 h. The reaction was quenched by an equal volume of CH_3_OH. After centrifugation at 13,000×*g* for 10 min, the mixture was taken into the bottle and detected with HPLC at 250 nm. HPLC detection procedure: 15% to 30% CH_3_CN in H_2_O from 0 to 5 min, 30% to 60% CH_3_CN in H_2_O from 5 to 10 min, 60% to 80% CH_3_CN in H_2_O from 10 to 15 min, 80% CH_3_CN in H_2_O from 15 to 18 min, 80% to 15% CH_3_CN in H_2_O from 18 to 20 min, 15% CH_3_CN in H_2_O from 20 to 28 min (H_2_O and CH_3_CN containing 0.1% TFA). The flow rate of 1 mL/min was used.

### Antagonistic activity assays

The antibacterial activity 1-hydroxyphenazine and phenazine-1-carboxylic acid was tested by paper-disc diffusion assay against five plant pathogenic fungi, *Bipolaris maydis, Alternaria* *solani, Fusarium* *graminearum, Phytophthora parasitica, Aspergillus flavus.* Paper discs of 5 mm diameter were autoclaved at 121 °C for 20 min. On this paper discs, 1-hydroxyphenazine and phenazine-1-carboxylic acid (dissolved CH_2_Cl_2_) was slowly applied in sterile conditions and the concentrations was 40 μg/disc. Paper discs with CH_2_Cl_2_ alone were used as control. The plates were incubated at 30 °C. These discs were then used to check the antibacterial activity of 1-hydroxyphenazine and phenazine-1-carboxylic acid.

### Fed-Batch fermentation

Using KB medium for seed culture, and then inoculating 500 μL of seed into 50 mL of KB medium in a 250 mL triplicate baffled Erlenmeyer flasks at 30 °C with 200 rpm for 12 h. The 50 mL seed culture was added into a 5 L fermenter containing 2 L PPM medium at 30 °C with an airflow rate of 3 L/min. The dissolved oxygen was kept at 30% by adjusting the agitation speed from 300 to 600 rpm. When the glucose is exhausted, the solution containing 500 g/L glucose was added into the fermenter at 2 mL/h and maintained 0.5–1 g/L of glucose.

## Supplementary Information

**Additional file 1. ** Tables of list of strains, plasmids (**Table S1**), primers (**Table S2**), comparison of *P. chlororaphis* H18 with GP72 (**Table S3**) and DNA sequences of *phzS* (**Table S4**). Antagonistic activity of phenazine-1-carboxylic acid and 1-hydroxyphenazine against plant pathogenic fungi (**Table S5**). And figures of PhzS mutation (**Figure S1** and **Figure S2**), Generation of mutant for *P. chlororaphis* H18 (**Figure S3**) and schematic of engineering strains of *P. chlororaphis* H18 for 1-OH-PHZ production (**Figure S4**). ^1^ H NMR and ^13^C NMR spectra of 1-hydroxyphenazine (**Figure S5** and **Figure S6**).

## Abbreviations

1-OH-PHZ: 1-Hydroxyphenazine
PCA: Phenazine-1-carboxylic acid
PEP: Phosphoenolpyruvate
PCN: Phenazine-1-carboxamide
2-OH-PHZ: 2-Hydroxyphenazine
TLC: Thin-layer chromatography
PTLC: Preparative thin-layer chromatography
