# Supplementary material for: Biosynthesis and metabolic engineering of 1-hydroxyphenazine in Pseudomonas chlororaphis H18
Source: Microb Cell Fact. 2021 Dec 30;20:235. doi: 10.1186/s12934-021-01731-y (PMC8717658; doi:10.1186/s12934-021-01731-y)
Supplement: Supplementary file 1 — Additional file 1. Tables of list of strains, plasmids (Table S1), primers (Table S2), comparison of P. chlororaphis H18 with GP72 (Table S3) and DNA sequences of phzS (Table S4). Antagonistic activity of phenazine-1-carboxylic acid and 1-hydroxyphenazine against plant pathogenic fungi (Table S5). And figures of PhzS mutation (Figure S1 and Figure S2), Generation of mutant for P. chlororaphis H18 (Figure S3) and schematic of engineering strains of P. chlororaphis H18 for 1-OH-PHZ production (Figure S4). 1 H NMR and 13C NMR spectra of 1-hydroxyphenazine (Figure S5 and Figure S6). [file 12934_2021_1731_MOESM1_ESM.docx]

**Supplementary Materials**

**Biosynthesis and metabolic engineering of 1-Hydroxyphenazine in *Pseudomonas chlororaphis* H18**

Yupeng Wan^1,2^, Hongchen Liu^1^, Mo Xian^1,2^* and Wei Huang^1,2^*

**Table S1.** Strains and plasmids used in this study ^a^

| Strains and Plasmids | relevant gene type | reference/ source |
| --- | --- | --- |
| Strains |  |  |
| DH5α | *E. coli* F-φ80*lac*ZΔM15 Δ(*lac*ZYA-*arg*F) U169 *end*A1 *rec*A1 *hsd*R17 (r_k_^-^ m_k_^-^) *sup*E44λ-thi-1 *gyr*A96 *rel*A1 *pho*A. | Lab stock |
| S17-1 (λpir) | *E. coli* res^-^ pro mod^+^ integrated copy of RP4, mob^+^, Donor strain for conjugation. | Lab stock |
| Rosetta (DE3) | F^-^*omp*T *hsd*S_B_(r_B_^-^m_B_^-^) *gal* *dcm*(DE3) pRARE(*arg*U, *arg*W, *ile*x, *gly*T, *leu*W, *pro*L) (Cm^r^) | Lab stock |
| BL21 (DE3) | F^-^ *omp*T *hsd*S_B_(r_B_^-^m_B_^-^) *gal dcm*(DE3), host strain for pET30a (+) | Lab stock |
| PH18 | *P. chlororaphis* H18 wild-type. | This study |
| PH18-1 | *phzO* was replaced by gene *phzS*. | This study |
| PH18-1-2 | *phzO* was replaced by mutant *phzS.* | This study |
| PH18-1-3 | *phzO* was replaced by two copies mutant *phzS.* | This study |
| PH18-1-4 | *rpeA* was replaced by gene *aroE* in PH18-1-3. | This study |
| PH18-1-5 | *rsmE* was replaced by gene *tktA* in PH18-1-4. | This study |
| PH18-1-6 | *psrA* was replaced by gene *aroB* in PH18-1-5. | This study |
| PH18-1-7 | *lon* was replaced by gene *ppsA* in PH18-1-6. | This study |
| PH18-1-8 | *prnA* was replaced by gene *gacA* in PH18-1-7. | This study |
| Plasmids |  |  |
| pK18mobsacB | Broad-host-range gene replacement vector, sacB, Kan^r^ | Lab stock |
| pK18-*phzS* | pK18mobsacB containing the *phzO* flanking region and gene *phzS* with its promotor, Kan^r^ | This study |
| pK18-*aroE* | pK18mobsacB containing the *rpe*A flanking region and gene *aroE*, Kan^r^. | This study |
| pK18-*tktA* | pK18mobsacB containing the *rsmE* flanking region and gene *tktA*, Kan^r^. | This study |
| pK18-*aroB* | pK18mobsacB containing the *psrA* flanking region and gene *aroB*, Kan^r^. | This study |
| pK18-*ppsA* | pK18mobsacB containing the *lon* flanking region and gene *ppsA*, Kan^r^. | This study |
| pK18-*gacA* | pK18mobsacB containing the *prnA* flanking region and gene *gacA*, Kan^r^. | This study |
| a Cm^r^ and Kan^r^ represent chloromycetin and kanamycin resistance, respectively. | | |

**Table S2.** Primers used in this work

| Application | Oligos and sequence(5’ to 3’) | application |
| --- | --- | --- |
| 16 S rRNA (27F) | AGAGTTTGATCCTGGCTCAG | Strain identification |
| 16 S rRNA (1492R) | GGTTACCTTGTTACGACTT |  |
| *phzS-F1* | TAAGGATCCATGAGCGAACCCATCGATATC | *phzS* alanine scanning |
| *phzS-R2* | TAACTCGAGCTAGCGTGGCCGTTCCACCTG |  |
| *phzS N48A-*R1 | GGCTGGATCGCGATGCCGACGCCAAGGGG |  |
| *phzS N48A-*F2 | TCGGCATCGCGATCCAGCCGGCGGCGGTCG |  |
| *phzS L76A-*R1 | ATGTAGCGCGCCTCGTGGGTGGGGATGGCG |  |
| *phzS L76A-*F2 | CCCACGAGGCGCGCTACATCGACCAGAGCG |  |
| *phzS M205A-*R1 | GCGACGATCGCGGTCTTGCCGTCGAGGAAG |  |
| *phzS M205A-*F2 | GCAAGACCGCGATCGTCGCCAACGACGAGC |  |
| *phzS V207A-*R1 | TCGTTGGCCGCGATCATGGTCTTGCCGTCG |  |
| *phzS V207A-*F2 | CCATGATCGCGGCCAACGACGAGCACTGG |  |
| *phzS R215A-*R1 | GCGACCAGCGCCGACCAGTGCTCGTCGTTG |  |
| *phzS R215A-*F2 | ACTGGTCGGCGCTGGTCGCCTATCCGATC |  |
| *phzS V217A-*R1 | GGATAGGCCGCCAGGCGCGACCAGTGCTCG |  |
| *phzS V217A-*F2 | CGCGCCTGGCGGCCTATCCGATCTCGGCGC |  |
| *phzS Y219A-*R1 | GAGATCGGCGCGGCGACCAGGCGCGACCAG |  |
| *phzS Y219A-*F2 | TGGTCGCCGCGCCGATCTCGGCGCGTCAC |  |
| *phzS P317A-*R1 | GCGCCCATCGCATACATCAGGTGGGCGGCG |  |
| *phzS P317A*-F2 | TGATGTATGCGATGGGCGCCAACGGCGCTT |  |
| *phzS M318A-*R1 | CTCGGCACCGCGCACACCCAGTTCACCAGC |  |
| *phzS M318A-*F2 | GGGTGTGCGCGGTGCCGAGCGCCGCCGTCG |  |
| *phzS G319A-*R1 | CCGTTGGCCGCCATCGGATACATCAGGTGG |  |
| *phzS G319A-*F2 | ATCCGATGGCGGCCAACGGCGCTTCGCAAG |  |
| *phzS M318T-*R1 | TTGGCGCCGGTCGGATACATCAGGTGGGCG | *phzS* key mutation site |
| *phzS M318T-*F2 | TGTATCCGACCGGCGCCAACGGCGCTTCGC |  |
| *phzS V217I-*R1 | GGATAGGCAATCAGGCGCGACCAGTGCTCG |  |
| *phzS V217I-*F2 | CGCGCCTGATTGCCTATCCGATCTCGGCGC |  |
| *phzS V207I-*R1 | TCGTTGGCAATGATCATGGTCTTGCCGTCG |  |
| *phzS V207I-*F2 | CCATGATCATTGCCAACGACGAGCACTGG |  |
| *aroE-*F | TAAGGATCCTCGAGTTCAACCTGGTCTA | Replacement of *rpeA* with *aroE.* |
| *aroE-*R | TAATCTAGACGTCGACGATAATACGGC |  |
| *rpeA-*LF | TAAGAATTCCTATCCGAATGGAGACTGCGC |  |
| *rpeA-*LR | TAAGGATCCGACCCTGACCGTCGAGGCCTC |  |
| *rpeA-*RF | TAATCTAGACTGAACGAGACGATGGTCACC |  |
| *rpeA-*RR | TAAAAGCTTGGCCTTGTCTTCGATCAACTG |  |
| *tktA-*F | TAAGGATCCGCCAGCTCCATTGTCATC | Replacement of *rsmE* with *tktA.* |
| *tktA-*R | TAATCTAGAGCCAGCAGTCTAGTGACG |  |
| *rsmE-*LF | TAAGAATTCGGCTGCCGCGGTGTTGTTG |  |
| *rsmE-*LR | TAAGGATCCCCGGACAAACGCGAAACAC |  |
| *rsmE-*RF | TAATCTAGAGATCGTGATGTCGTCACCG |  |
| *rsmE-*RR | TAAAAGCTTGCAATCCACCGAGTTCTAC |  |
| *aroB-*F | TAAGGATCCCATGCCTCGGTGGAGCAGCAG | Replacement of *psrA* with *aroB.* |
| *aroB-*R | TAATCTAGACTGTAGCGCGCCAGATGGTGC |  |
| *psrA-*LF | TAAGAATTCGTCCTGGCTCAGTTCCCTGAG |  |
| *psrA-*LR | TAAGGATCCGAACAATTGCTCGGCAGCATC |  |
| *psrA-*RF | TAATCTAGAATGGCTGCGGCGCAACTCAAG |  |
| *psrA-*RR | TAAAAGCTTGATAGTTCACCTGATGCGGCG |  |
| *ppsA-*F | TAAGGATCCCGCTGTCGATATACGGCCGC | Replacement of *lon* with *ppsA.* |
| *ppsA-*R | TAATCTAGACGGCTTCCAGATGGAGCCCG |  |
| *lon-*LF | TAAGAATTCGGCTGAGTGCTGAACAGTTCC |  |
| *lon -*LR | TAAGGATCCCGGCGTTTTCCGGGTCGGCG |  |
| *lon -*RF | TAATCTAGAGGCATCACCGTGCACTTCGC |  |
| *lon -*RR | TAAAAGCTTCCGAGCCCGCGAGGCTGCGC |  |
| *gacA-*F | TAAGGATCCCGAACATGCGATACACGCCAG | Replacement of *prnA* with *gacA.* |
| *gacA-*R | TAATCTAGAAGGACGCGATCCGGCACCTG |  |
| *prnA-*LF | TAAGAATTCCGATAAGCCTGGTCGTCCG |  |
| *prnA-*LR | TAAGGATCCGACTACCTGCGGTCGCTGC |  |
| *prnA-*RF | TAATCTAGAGACGAGGTACGAGGCGGCCATC |  |
| *prnA-*RR | TAAAAGCTTGATATCTATTCCGGCACCAGC |  |

**Table S3.** Comparison of *P. chlororaphis* H18 core loci *phzABCDEFGO* with *Pseudomonas chlororaphis* GP72

| ID | Core *Phz* genes | Function | #aa | Per. Ident |
| --- | --- | --- | --- | --- |
| 1 | *phzI* | acyl-homoserine-lactone synthase | 196 | 97.96% |
| 2 | *phzR* | transcriptional regulator | 245 | 98.78% |
| 3 | *phzA* | ketosteroid isomerase | 163 | 100% |
| 4 | *phzB* | nuclear transport factor 2 | 163 | 98.77% |
| 5 | *phzC* | phospho-2-dehydro-3- deoxyheptonate aldolase | 400 | 99.75% |
| 6 | *phzD* | isochorismatase | 207 | 100% |
| 7 | *phzE* | anthranilate synthase | 636 | 99.84% |
| 8 | *phzF* | 2,3-dihydro-3-hydroxyanthranilate isomerase | 278 | 99.64% |
| 9 | *phzG* | FMN-dependent oxidase | 222 | 99.10% |
| 10 | *phzO* | aromatic monooxygenases | 491 | 99.59% |

**Table S4.** DNA sequences of *phzS* and its promoter from *Pseudomonas aeruginosa* PAO1

| Gene | DNA sequences |
| --- | --- |
| P*_phzS_* | CGACACCGCTGCGCCGGCGTTTCATGGCGGATAACCGCAAGCGGTTATTCGCCCTACGCGGCCTTGGAGCCCATCTAACCGCACGCGGTCATGCGTACCGCGGCCTCGGAGCCGGTTCGTAGGGCGAATGACGCCACCGGCGTTATCCGCCGCTGCGCCGACGTTTCATCGCGGTAAACGGTCATCCATCCCAGCCGAACCCCCATCGATTCGAACACTCGAGAAAAGGAAGCACCC |
| *phzS* | **ATG**AGCGAACCCATCGATATCCTCATCGCCGGCGCCGGCATCGGCGGCCTCAGTTGCGCCCTGGCCCTGCACCAGGCCGGCATCGGCAAGGTCACGCTGCTGGAAAGCAGCAGCGAGATACGCCCCCTTGGCGTCGGCATCAATATCCAGCCGGCGGCGGTCGAGGCCCTTGCCGAACTGGGCCTCGGCCCGGCGCTGGCGGCCACCGCCATCCCCACCCACGAGCTGCGCTACATCGACCAGAGCGGCGCCACGGTATGGTCCGAGCCGCGCGGGGTGGAAGCCGGCAACGCCTATCCGCAGTACTCGATCCATCGCGGCGAACTGCAGATGATCCTGCTCGCCGCGGTGCGCGAGCGCCTCGGCCAACAGGCGGTACGCACCGGTCTCGGCGTGGAGCGTATCGAGGAGCGCGACGGCCGCGTGCTGATCGGCGCCCGCGACGGACACGGCAAGCCCCAGGCGCTCGGTGCCGATGTGCTGGTCGGCGCCGACGGTATCCATTCGGCGGTCCGCGCGCACCTGCATCCCGACCAGAGGCCGCTGTCCCACGGTGGGATCACCATGTGGCGCGGCGTCACCGAGTTCGACCGCTTCCTCGACGGCAAGACCATGATCGTCGCCAACGACGAGCACTGGTCGCGCCTGGTCGCCTATCCGATCTCGGCGCGTCACGCGGCCGAAGGCAAGTCGCTGGTGAACTGGGTGTGCATGGTGCCGAGCGCCGCCGTCGGCCAGCTCGACAACGAGGCCGACTGGAACCGCGACGGGCGCCTGGAGGACGTGCTGCCGTTCTTCGCCGACTGGGACCTGGGCTGGTTCGACATCCGCGACCTGCTGACCCGCAACCAGTTGATCCTGCAGTACCCGATGGTAGACCGCGATCCGCTGCCGCACTGGGGCCGGGGACGCATCACCCTGCTCGGCGACGCCGCCCACCTGATGTATCCGATGGGCGCCAACGGCGCTTCGCAAGCAATCCTCGACGGCATCGAGCTGGCCGCCGCGCTGGCGCGCAACGCCGACGTGGCCGCAGCCCTGCGCGAATACGAAGAAGCGCGGCGGCCGACCGCCAACAAGATCATCCTGGCCAACCGAGAACGGGAAAAAGAGGAATGGGCCGCGGCTTCGCGACCGAAGACCGAGAAGAGCGCGGCGCTGGAAGCGATCACCGGCAGCTACCGCAACCAGGTGGAACGGCCACGC**TAG** |


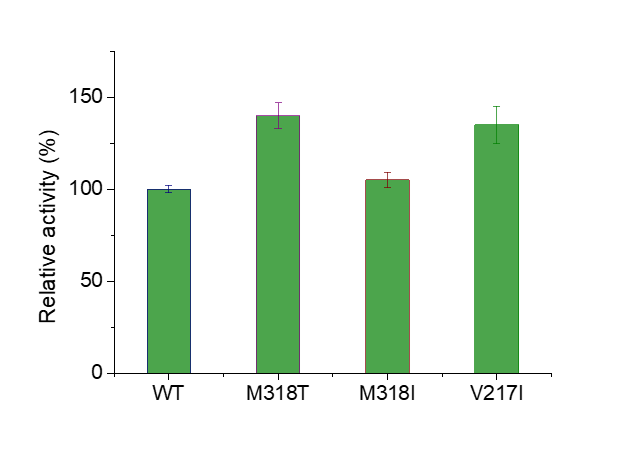


**Fig. S1** Relative activity of PhzS^M318T^, PhzS^M318I^, PhzS^V217I^ and wild type. The data are from three experiment replicates, and are expressed as the mean value ± SD.

**Fig. S2** The relative activity of PhzS wild-type and M205, V207, R215, P317, G319 mutated to I, S, V, and T. The data are from three experiment replicates, and are expressed as the mean value ± SD.


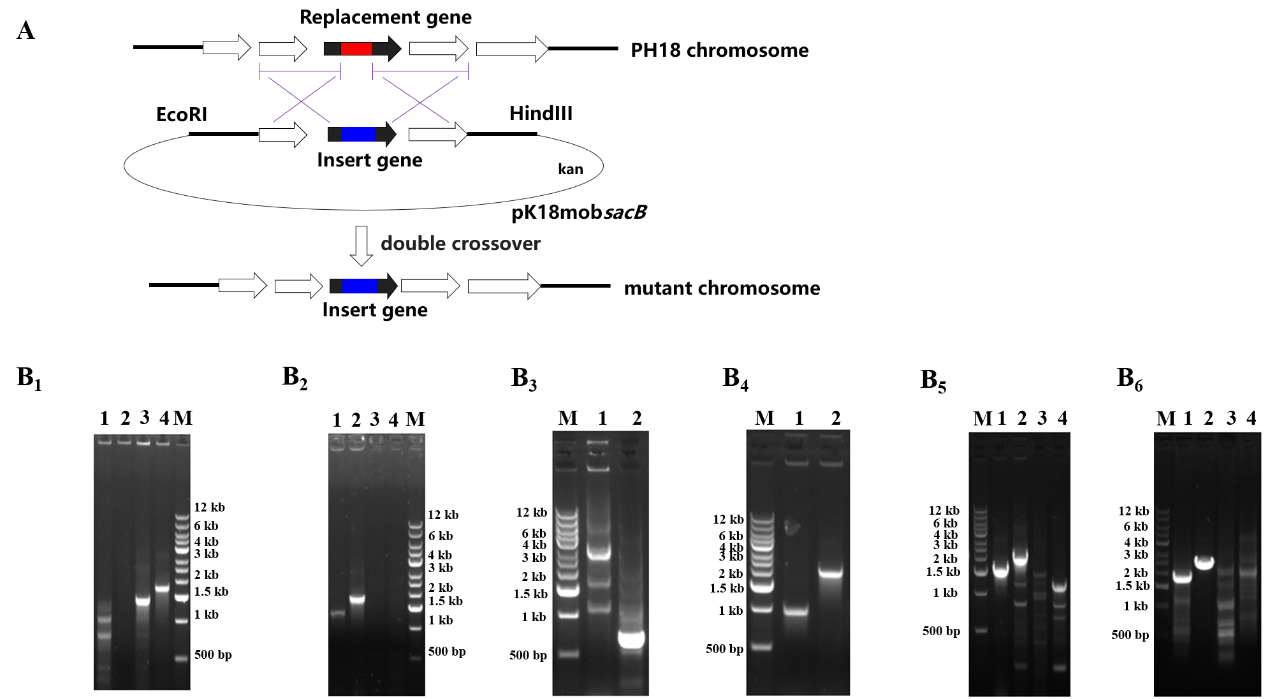


**Fig. S3** Generation of mutant for *P. chlororaphis* H18*.* **A** Schematic diagram of the principle and general steps of gene replacement in *P. chlororaphis* H18. **B_1_** Diagnosis of *phzS* replacement of *phzO* by PCR assay. M, marker; 1, 2, *P. chlororaphis* H18 without any banding; 3, 4, PH18-1 mutant with the expected size of 1501 bp and 1780 bp. **B_2_** Diagnosis of *aroE* replacement of *rpeA* by PCR assay. M, marker; 1, 2, PH18-1-4 mutant with the expected size of 1348 bp and 1787 bp; 3, 4, *P. chlororaphis* H18 without any banding. **B_3_** Diagnosis of *tktA* replacement of *rsmE* by PCR assay. M, marker; 1, PH18-1-5 mutant with the expected size of 2946 bp; 2, *P. chlororaphis* H18 with the size of 765 bp. **B_4_** Diagnosis of *aroB* replacement of *psrA* by PCR assay. M, marker; 1, *P. chlororaphis* H18 with the size of 1074 bp; 2, PH18-1-6 mutant with the expected size of 1992 bp; **B_5_** Diagnosis of *ppsA* replacement of *lon* by PCR assay. M, marker; 1, 2, PH18-1-7 mutant with the expected size of 1606 bp and 2096 bp; 3, 4, *P. chlororaphis* H18 without any banding. **B_6_** Diagnosis of *gacA* replacement of *prnA* by PCR assay. M, marker; 1, 2, PH18-1-8 mutant with the expected size of 1710 bp and 2325 bp; 3, 4, *P. chlororaphis* H18 without any banding.


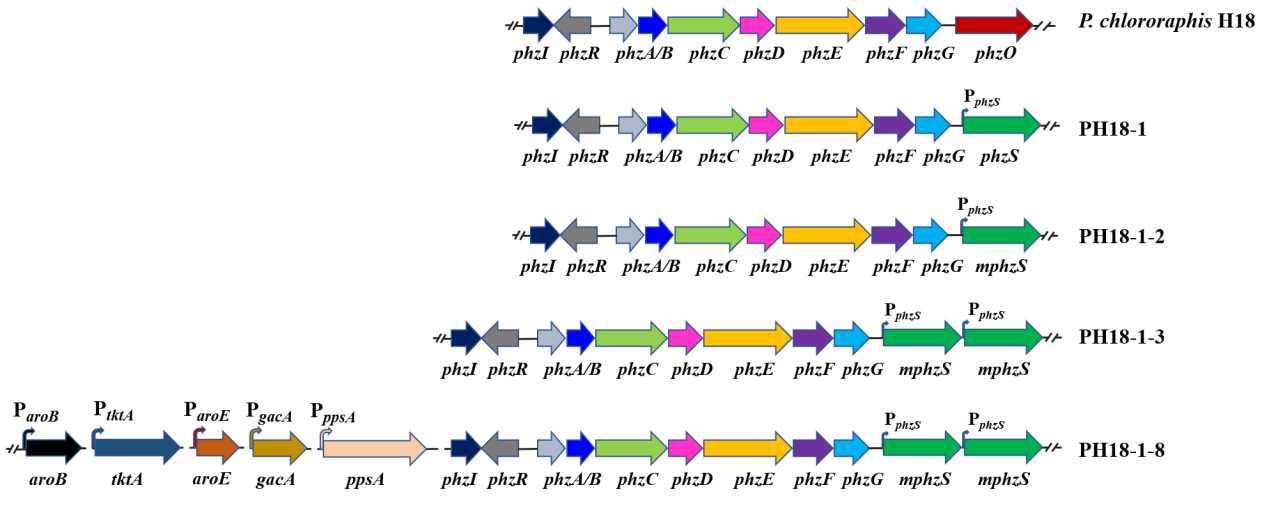


**Fig. S4** Schematic of engineering strains of *P. chlororaphis* H18 for 1-OH-PHZ production. PH18-1: PH18::P*_phzS_phzS*Δ*phzO*; PH18-1-2: PH18::P*_phzS_mphzS*Δ*phzO;* PH18-1-3: PH18::P*_phzS_*2*mphzS*Δ*phzO;* PH18-1-8: PH18::P*_phzS_*2*mphzS*::P*_aroE_aroE*::P*_tktA_tktA*::P*_aroB_aroB*::P*_ppsA_*

*ppsA*::P*_gacA_gacA*Δ*phzO*Δ*rpeA*Δ*rsmE*Δ*psrA*Δ*lon*Δ*prnA*; *mphzS*: PhzS^M318A V207I^.

**Table S5.** Antagonistic activity of phenazine-1-carboxylic acid and 1-hydroxyphenazine against plant pathogenic fungi.

| Plant pathogenic fungi | ^a,b^Inhibition zone (mm) ± SE by | |
| --- | --- | --- |
|  | phenazine-1-carboxylic acid | 1-Hydroxyphenazine |
| *Bipolaris maydis* | 8±0.26 | 12±0.11 |
| *Alternaria* *solani* | 3±0.35 | 14±0.32 |
| Fusarium graminearum | 4±0.16 | 32±0.36 |
| Phytophthora parasitica | 18±0.52 | 6±0.21 |
| *Aspergillus flavus* | 2±0.12 | 8±0.33 |

^a^After 4 days of inoculation; ^b^Average of 3 replications.


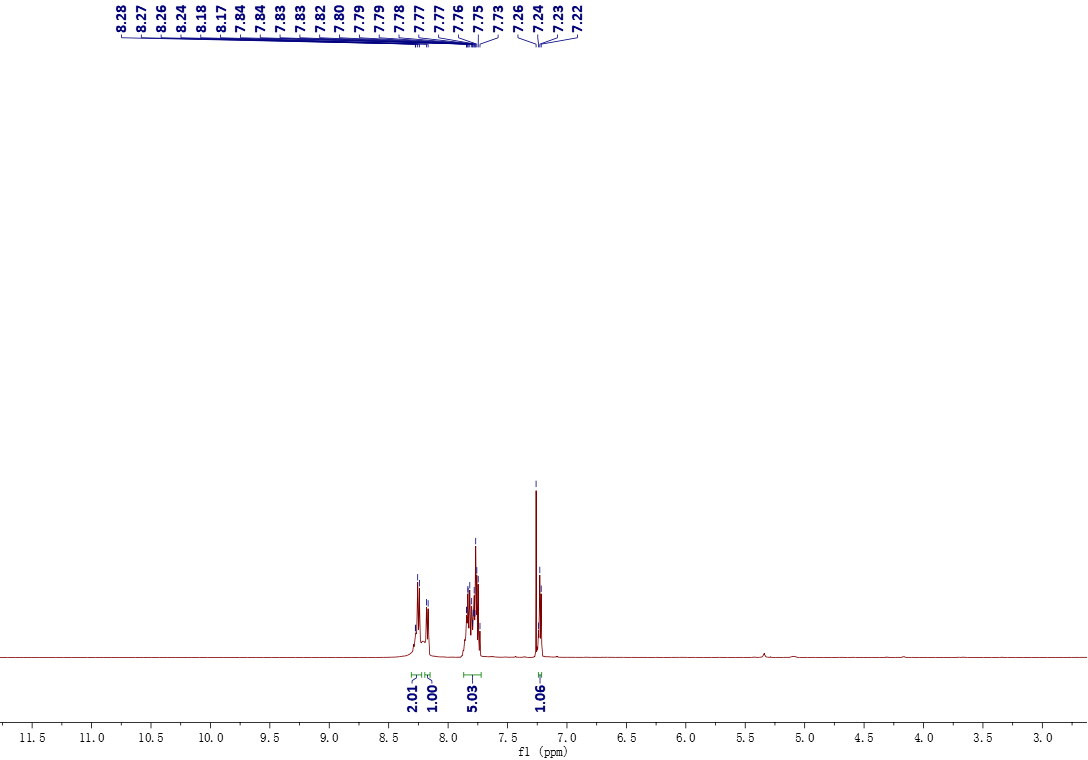


**Fig. S5** ^1^ H NMR (600 MHz, CDCl_3_) spectra of 1-hydroxyphenazine.


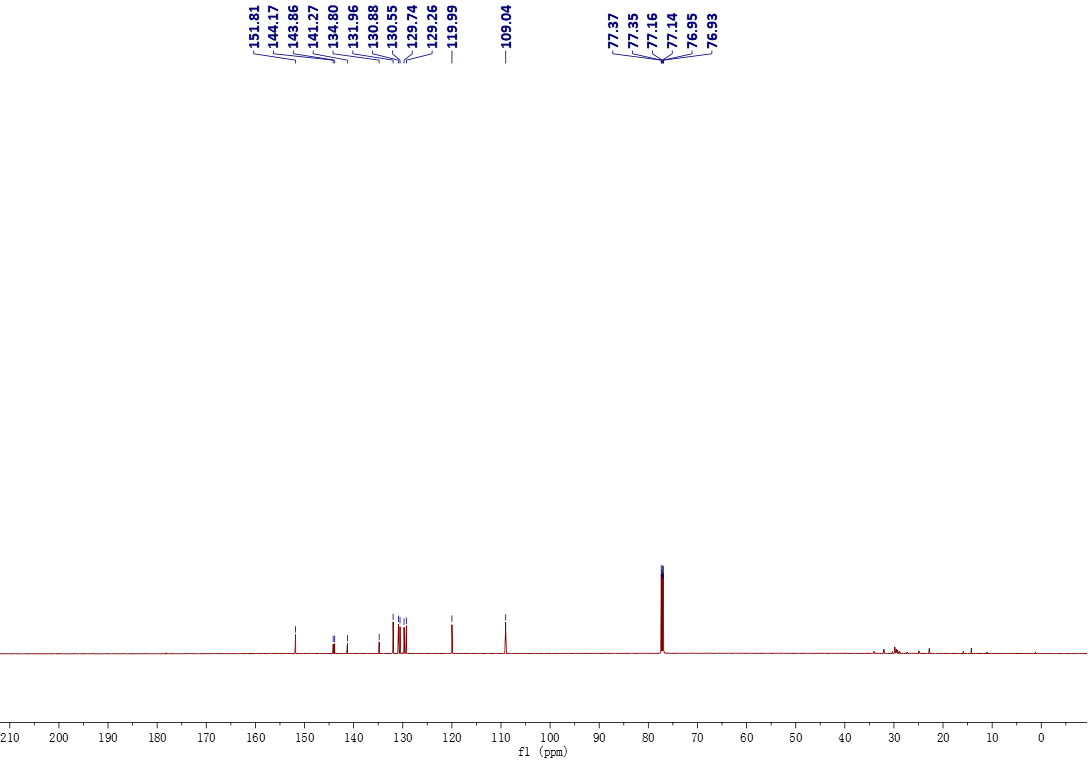


**Fig. S6** ^13^C NMR (150 MHz, CDCl_3_) spectra of 1-hydroxyphenazine.
